# Recent Trends in Imaging for Atrial Fibrillation Ablation

**Published:** 2010-05-05

**Authors:** Rajesh Kabra, Jagmeet Singh

**Affiliations:** Cardiac Arrhythmia Service, Massachusetts General Hospital Heart Center, Harvard Medical School, Boston, Massachusetts, USA

**Keywords:** Atrial fibrillation, catheter ablation, imaging

## Abstract

Catheter ablation provides an important treatment option for patients with both paroxysmal and persistent atrial fibrillation. It mainly involves pulmonary vein isolation and additional ablations in the left atrium in persistent cases. There have been significant advancements in this procedure to enhance the safety and effectiveness. One of them is the evolution of various imaging modalities to facilitate better visualization of the complex left atrial anatomy and the pulmonary veins in order to deliver the lesions accurately. In this article, we review the electroanatomic mapping systems including the magnetic-based and impedence-based systems. Each of these mapping systems has its own advantages and disadvantages. In addition, we also discuss the role of intracardiac echocardiography and three dimensional rotational angiography in atrial fibrillation ablation.

##  Introduction

Over the past decade, catheter ablation has emerged as an important therapeutic strategy for the management of both paroxysmal and persistent atrial fibrillation (AF). The contemporary approach to catheter ablation of AF is based on two fundamental principles: (1) isolation of the pulmonary veins and other venous structures (e.g. superior vena cava etc.) to eliminate the triggers for AF and (2) substrate modification to eliminate the ability of the atria to sustain AF. Substrate modification approach at this time is usually reserved for patients with persistent AF, as this subgroup has a significantly more enlarged and remodeled atrium.

The most widely used  ablation strategy involves creating a lesion set encompassing a wide circumferential area around the pulmonary veins (PV) (i.e. involving the antrum) with subsequent verification of conduction block. In patients with persistent AF, long term success can be further enhanced by incorporating adjunctive ablation strategies to PV isolation, such as targeting sites demonstrating complex atrial fractionated electrograms (CFAEs) during AF or creating linear lesion sets over the left atrial roof, posterior wall, mitral isthmus or cavo-tricuspid isthmus. These strategies require a firm understanding of the anatomy of the left atrium (LA) and the pulmonary veins. While a majority of patients have four PVs, two superior and two inferior with independent ostia draining separately into the LA, a number of variations have been described in the literature [1,2].  Based on one study, cardiac MRI identified a variant pulmonary vein anatomy in up to 38% of the cases [3]. These include left common ostium (commonest), right common ostium, and a separate origin for the right middle PV. Although prior knowledge of the diameter of the PVs is useful in the sizing of circular mapping catheters, it is essential if balloon-based ablation catheters are to be used, as a close approximation of the balloon to the PV ostia is required for successful isolation. The anatomic variations in the left atrial ridges and pouches, the unpredictable numbers of veins, inconsistent ostial sizes, and variable take-offs and locations can significantly impact procedural success [4]. In addition, the knowledge of the anatomical location of surrounding structures like esophagus, aorta with respect to the left atrium is important in order to prevent any inadvertent complications [5].

Conventional fluoroscopy is uniformly used for real time visualization of all the catheters. However, it provides only two-dimensional representation of any image. In addition, the anatomic variations are inadequately recognized, and thereby can lead to prolonged procedural times, increased radiation exposure and imprecise lesion delivery. These limitations have been markedly reduced through technological advancements resulting in the usage of computer based electro anatomic mapping systems, intracardiac echocardiogram, rotational angiography as well as three dimensional echocardiogram.

In this article, we provide a brief overview of the commonly used electroanatomic mapping systems in the electrophysiology laboratories. These systems use either a magnetic field or an electrical impedance field to localize the catheters within the heart. This article describes and compares these modalities, highlighting their advantages and limitations with respect to mapping and ablation. In addition we will also discuss the role of intracardiac echocardiogram and 3D rotational angiography and their application in AF ablation.

## Magnetic Electroanatomic Mapping System

The use of the magnetic electroanatomic mapping system (CARTO, Biosense-Webster Inc, Diamond Bar, CA) was initially described by Ben-Haim et al in 1997 [6]. It is based on the principle that a coil placed in the magnetic field will generate an electrical current. The magnitude of the current depends on the orientation of the coil and the strength of the magnetic field. In the electrophysiology laboratory, the magnetic field is generated by a unit mounted under the patient table (5 x 10^-6^ to 5 x 10^-5^ Tesla) which creates the mapping space around the patient's chest. The locator pad has three coils, each of which generates a magnetic field which decays as a function of distance from that coil. The sensor within the catheter measures the strength of the magnetic field which determines its distance from each coil. These distances are radii of the theoretical spheres around each coil and the intersection of all the three spheres helps determine the location and orientation of the sensor within the catheter tip.

## CARTO™

 This mapping system uses proprietary deflectable catheters which are available in various sizes (3.5 mm, 4 mm, 8mm) and may have either a solid or an irrigated tip. These catheters have a locator sensor at the distal end which send signals to the processing unit. A 3-D electroanatomic map of any cardiac chamber of interest can be created with these catheters using point by point mapping. Additionally, the local electrograms at each point can be gated to a preselected reference electrogram to create activation or propagation color-coded maps as well as a voltage map that can be superimposed on the anatomical map of the chamber. While there is no fixed reference electrogram during atrial fibrillation, as there is fluctuating cycle length both in atria as well as ventricle, during post AF ablation atrial tachycardia and atrial flutter, reference electrogram timing becomes critical. Generally, the atrial electrograms of the CS catheters are used as reference, given their stability and prominent atrial signals. For focal and microreentrant atrial tachycardias, the area of earliest activation is surrounded uniformly by areas of gradually later activation. These central areas of earliest activation are good targets for ablation. On the other hand, with macroreentrant atrial arrhythmias, areas of earliest activation meet the areas of latest activation.  In these arrhythmias, ablation should be performed across a narrow isthmus and not necessarily in the area of earliest activation. These maps can be viewed in multiple projections. Areas of interest like the His bundle, scars, fractionated electrograms, ablation points, or points close to the esophagus or phrenic nerve can be tagged in these maps. A reference patch is placed over the patient's back to correct for minor patient movement. One limitation of the CARTO™ system was that the proprietary ablation catheter uses only a single bipolar electrode in the catheter tip to record electroanatomic data. Therefore, it can be time consuming to generate a high-density electroanatomic map of a chamber. The need for point-to-point mapping on a beat-by-beat basis limits the use of this mapping system for non-sustained or unstable rhythms.

## CARTO-3™

**Figure 1:**
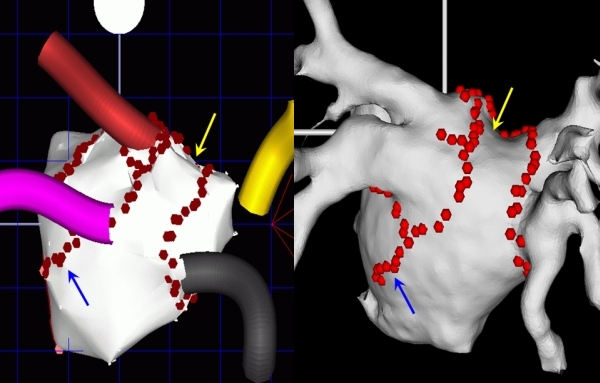
The electroanatomic map of the left atrium using CARTO (left) can be integrated with the CT/MRI images using CARTOMERGE Module (right). The arrows depict the corresponding points on the two maps.

A significant advancement in this technology was development of a hybrid magnetic and current based mapping system (CARTO 3™). CARTO 3™ System is the third generation platform from Biosense Webster where multiple catheter tips and curves can be visualized on the electroanatomic map. This uses an Advanced Catheter Location (ACL) technology which is a hybrid of the old magnetic location with current based localization of the catheters. A magnetic field is generated by 3 coils. A location sensor in the catheter measures the strength of the field and the distance from each coil. The location of the sensor and the catheter is determined by the intersection of the three fields. In addition to this magnetic field, CARTO 3™ uses an electrical field created by two sets of patches. The magnetic technology calibrates the current-based technology thereby, minimizing distortions at the periphery of the electrical field. The system generates a small current that is sent from the electrodes of the catheters to six patches on the patient's body. Each electrode emits current at a unique frequency. The strength of the current emitted by each electrode is measured at each patch and creates a 'current ratio' which is unique to each electrode's location. Mapping is performed in two steps. Initially, the magnetic mapping permits precise localization of the catheter with the sensor. This is associated with the current-ratio of the electrode closest to the sensor. As the catheter with the sensor moves around a chamber, multiple locations are created and stored by the system. The system now integrates the current based points with their respective magnetic locations resulting in a calibrated current-based field, which permits accurate visualization of catheters and their locations. Each electrode emits a unique frequency that provides clear distinction of the electrodes, especially when they are close to each other. Both the catheters with and without the magnetic sensors can be visualized without spatial distortions. This system has 'Fast Anatomical Mapping' (FAM) feature that permits rapid creation of anatomical maps by movement of sensor based catheter. Unlike point-by-point electroanatomical mapping, volume data can be collected with FAM. Catheters besides the ablation catheter such as the multi-polar Lasso, can further enhance the collection of points and increase the mapping speed. The CARTO-3™ system provides a highly accurate geometry of a cardiac chamber, which can be visualized in multiple views (Figure 1). Activation maps are straightforward, and respiratory artifact is limited.

##  CARTO-Merge®

**Figure 2:**
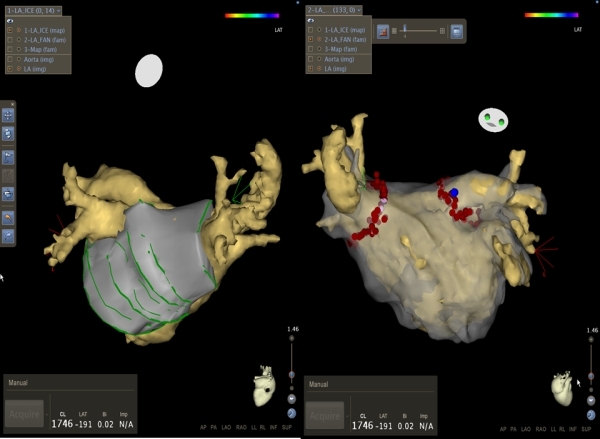
Use of intracardiac echocardiogram to facilitate image integration by CARTO 3. Left: Posterior view of the left atrium with overlay of images acquired by intracardiac echocardiogram. Right: Left atrial image following image integration with pre-acquired MRI image.

**Figure 3:**
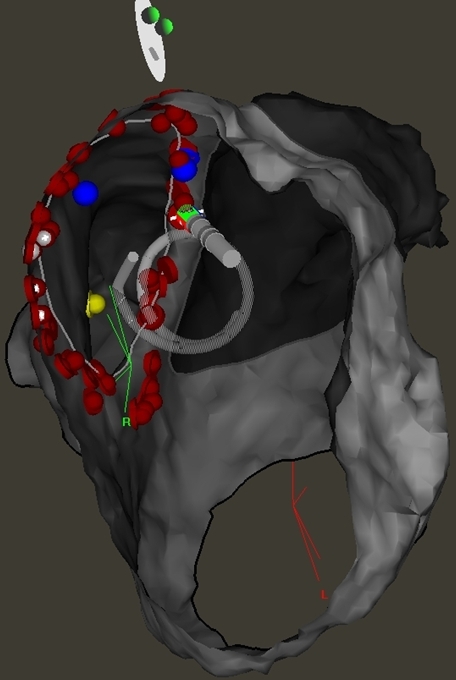
An internal view (right lateral) of the left atrium using CARTO demonstrates the ridge between the left atrial appendage and left pulmonary veins which is a common site of pulmonary vein reconnection.

CARTO-Merge® Module facilitates the identification of specific anatomical landmarks (i.e., pulmonary vein ostia) on the segmented CT or MRI image, as well as on the generated CARTO® map of the left atrium (LA). These images are then locked together to create the integrated image. Subsequently, the imagecan be further refined by obtaining more endocardial points all over the left atrium, to ensure uniform distribution over the entire anatomy. The adequacy of the integration can be confirmed by roving the catheter through the atrium and assessing its relationship to well-recognized landmarks (i.e., veins, ridges and appendage). CARTOMerge® helps to guide real-time catheter ablation using the detailed left atrial anatomy acquired from the CT/MRI [7,8]. These have also been used in atrial fibrillation ablation to correctly identify the anatomy and location of the pulmonary veins as well as other structures like the esophagus [9]. Studies have shown that they may improve the safety and efficacy of the procedure [10]. For the accurate registration, it is crucial to acquire CT/MRI images at end expiration at the level of the functional residual volume rather than the end-inspiratory state to prevent the superior-inferior displacement of the left atrium and the splaying-narrowing of the PVs with respiration [11].  The left atrium and pulmonary veins are mapped in the standard fashion to create an anatomical shell which is registered to the CT/MRI image by selecting corresponding fiducial points on the electroanatomic map and the CT/MRI image (Figure 1). The mapping system then provides an average tip-to-surface distance, which ideally should be less than 2 mm. During the catheter manipulation during ablation, the projected catheter distance to the surface is used as an additional guide to assess catheter contact. This can be complemented by information from electrograms from the catheter, fluoroscopy as well as intracardiac echocardiogram (Figure 2).  The orthogonal views of the registered images in the electroanatomic system help to identify the catheter position. The endoluminal view can be generated from these maps to provide excellent visualization of the ridge between the LA appendage and the left pulmonary veins, which is a common site for PV-LA reconnection (Figure 3). 

## CARTO-Sound

Similarly, the CartoSound™ Image Integration Module (Biosense Webster, Inc.) incorporates the electroanatomic map to a map derived from intracardiac echocardiography (ICE)),  which provides  an innovative strategy for 3-D reconstruction of the cardiac chambers using real-time ICE [12,13].The ICE catheter has an embedded navigation sensor and can be positioned either in the right atrium, coronary sinus or even at times across a transseptal puncture into the left atrium [14]. A 3-D volume rendered image is created by obtaining electrocardiogram-gated echocardiographic images of the endocardial surface of the left atrium. The utility of this strategy includes detailed visualization of the left atrium, its adjacent structures and elimination of chamber deformity, which often happens with contact mapping. The anatomical maps of the left atrium can then be registered to the patient's CT or MRI using two or more fiducial points. In our center, if the image quality from the right atrium is suboptimal, we occasionally cross to the left atrium through a pre-existing transseptal puncture, for better left atrial visualization. This enables better delineation of landmarks such as the mitral valve annulus, right-sided pulmonary vein ostia, and the ridge between the LA appendage and the left superior pulmonary vein ridge [14]. While this strategy is safe, a careful manipulation of the stiff  ICE catheter in the left atrium.

## EnSite NavX™ System Overview

**Figure 4:**
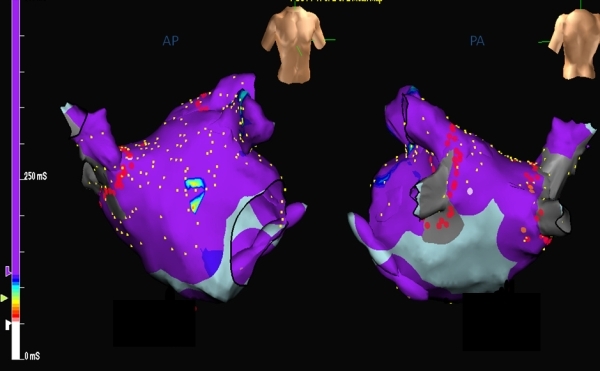
Left atrial electroanatomic map generated by Ensite NavX (Left: anterior view, Right: posterior view). Red dots represent the ablation points, while the yellow dots represent the points acquired by multielectrode mapping catheter.

**Figure 5:**
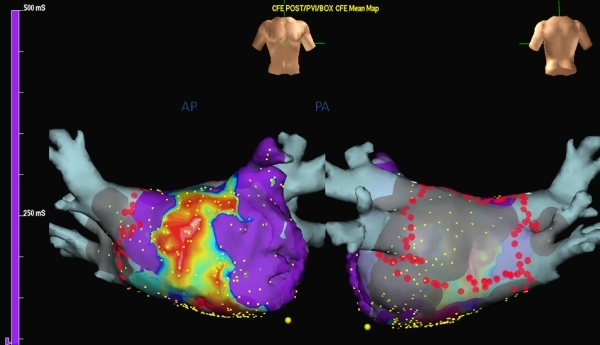
Representation of the complex fractionated atrial electrograms (CFAE) using Ensite NavX automated software. Left: Anterior view of the left atrium showing predominant location of CFAEs over anterior wall and the roof. Right: Posterior view of the left atrium showing the ablation lesion sets (red dots) for pulmonary vein isolation and the left atrial posterior wall.

EnSite NavX™ (St. Jude Medical, Inc., Minneapolis MN) is another commonly used mapping system for catheter ablation of atrial fibrillation [15,16]. The fundamental principle underlying EnSite NavX™ (St. Jude Medical, Inc., Minneapolis MN) navigation is that of an impedance-based measure, which is dependent on the voltage gradient that exists across tissue when a current is applied through the surface electrodes. This mapping system is based on localization of multiple electrodes using an electrical field generated by three pairs of surface electrodes placed on the patient's body along three orthogonal axes. The patches emit a low-current, high-frequency electrical field (5.7 kHz signal) across the chest using different frequencies for the x, y and z axes. The conventional catheters are localized by measuring the electrical potential or field strength received by them. The measured voltage and impedance drop sensed by the catheter electrodes are proportional to the distance of the electrodes from the patches. These measures are referenced to an electrode, also called the reference electrode, which is the origin of the coordinate system. The position of this reference intracavitary electrode needs to be stable in order to maintain the accurate position of the electroanatomic map. Any significant shift in its position can frequently lead to reinitiation of the mapping. This is commonly placed in the distal coronary sinus, which has advantages of catheter stability as well as concordant respiratory movements with the left atrium.  However, there are chances of dislodgement especially during ablation in coronary sinus during persistent atrial fibrillation case. The other options are high lateral right atrium, pulmonary artery, hepatic veins or even descending aorta. The conventional electrophysiology catheters can be used with this mapping system. The three dimensional location of each catheter electrode is located by sensed voltage gradients in all the axes. All the electrodes are displayed simultaneously as catheter bodies with real-time navigation, facilitating nonfluoroscopic navigation, mapping and creation of cardiac chambers. Any multielectrode catheter can be used to create a geometric model of a cardiac chamber (Figure 4).  This involves creating a cloud of points to represent the geometry of the cardiac chamber. At the completion of the point cloud, the final anatomy is displayed as a best-fit surface projected to the point cloud. A limitation of this approach is that there may be interpolations in the region of curvature that do not depict the accurate geometry. This is more prominent at areas of exvaginations like the pulmonary veins or the left atrial appendage. One strategy to minimize this is to create volumes of these structures in separate maps and then combining them to the main chamber. An electroanatomic map is generated by acquiring and displaying the activation and voltage data on this model. The ability for graphic display of multiple catheters can significantly reduce fluoroscopic time and facilitate complex atrial fibrillation ablations. The system also has the capability to import and integrate three-dimensional CT or MRI images to facilitate anatomically-based ablation procedures [17]. The system facilitates recording of points of ablation as well as other points of interest like esophageal temperature increases. Similar to the CARTO system, the voltage or the activation map can be superimposed on the geometry. Complex fractionated electrograms can be targeted in persistent atrial fibrillation using the software to depict the mean electrogram cycle length map (Figure 5).

With the use of multipolar catheters,dense electroanatomic maps and endocardial surface geometry can be generated in a short period of time. All the catheter electrodes can be visualized within the CT/MRI integrated cardiac geometry to assist the ablation procedure by providing 3-D information about the relative positions of the catheters within the heart. The EnSite NavX™ also provides timing and signal data, which are critical to assess the adequacy of lines created during the AF ablation. Both EnSite NavX™ as well as CARTO systems help in mapping  the flutter circuits, directing ablation of complex fractionated electrograms and facilitating targeted substrate ablation in patients with persistent atrial fibrillation.

NavX Fusion provides a significant advancement  in image integration with the EnSite NavX system [17], This technique has the ability to dynamically mold the created geometry into the CT image. After the left atrial geometry is created and appears similar to the CT image, a field scaling algorithm is applied. This adjusts for the non-linearity of the geometry and takes into account the measured inter-electrode spacing for all the locations within the geometry. After field scaling, the left atrial geometry is fused into the CT in two stages, termed as primary and secondary fusion. The primary fusion uses four fiducial corresponding landmarks on both the CT and the created geometry. These points lock both the images together, ensuring reasonable 3-D anatomic separation. At the sites of local mismatch between th two images, secondary fusion is applied to enable the molding process [17]. While this is a validated approach, it is still possible to have errors of integration.

**Figure 6:**
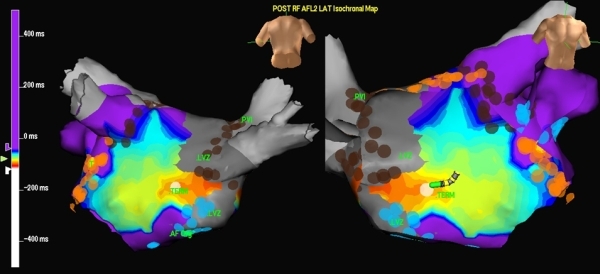
Activation mapping of the atrial flutter following pulmonary vein isolation using Ensite NavX. The figures (Left: posterior view of the left atrium and pulmonary veins, Right: anterior view of the left atrium) demonstrate ‘early meets late’ phenomena suggesting a macro-reentrant atrial flutter.

Both magnetic and impedence basded mapping systems provide the necessary anatomical information needed atrial fibrillation ablation. While they both have different algorithms, they enable the operator to generate complex fractionated electrogram maps and target the appropriate regions for ablation, generate an activation map to identify the circuits of atrial flutter (Figure 6). However, the data on a head-to-head comparison of these different electroanatomic mapping technologies on safety, efficacy and outcomes is still needed.

## Intracardiac Ultrasound

Intracardiac ultrasound provides a useful imaging tool for continuous direct visualization of all the chambers of the heart as well as the pulmonary veins during catheter ablation of atrial fibrillation. Over the years, there has been a significant improvement in the technology with the advent of low frequency (12.5 - 9 MHz) and more recently the phased array (5.5 - 10 MHz) transducers which have been miniaturized and mounted on the catheters capable of percutaneous insertion [18,19]. Phased array ICE imaging uses a 64-element transducer on the distal end of an 8 Fr or 10 Fr catheter. These catheters are capable of M-mode, pulsed, continuous wave and color Doppler. Their variable frequency, steerability and flexibility permits higher resolution, deeper penetration, and better quality images of all the cardiac chambers from the right atrium. These are advanced through the femoral venous sheaths under fluoroscopic guidance and positioned in the mid right atrium. Through clockwise rotation of the catheter one can sequentially visualize the interatrial septum, foramen ovale, left atrial appendage, left pulmonary veins, descending aorta, esophagus, posterior left atrial wall, right pulmonary veins. The catheter may also be advanced into the coronary sinus or the left atrium for better visualization of the left atrium and the pulmonary veins.

One of the main uses of the ICE imaging includes facilitation of transseptal puncture by guiding the needle to the membranous part of the fossa ovalis [20]. It also helps in identifying variants like atrial septal aneurysm and lipomatous hypertrophy. We routinely anticoagulate our patients prior to transseptal catheterization to prevent left atrial thrombus based on the increased security provided by ICE. Once transseptal access is achieved, ICE facilitates visualization of the left atrial and pulmonary venous anatomy. The number and position of the pulmonary veins openings can be determined to guide the pulmonary vein antrum ablation away from the pulmonary venous ostia to prevent pulmonary vein stenosis [21,22].  It also helps to assess the electrode-tissue contact. The images of ICE can be integrated with the electroanatomic mapping systems (CARTOSOUND) to generate the geometry of left atrium. A recent study demonstrated the feasibility of catheter ablation of atrial fibrillation without fluoroscopy using intracardiac echocardiography and electroanatomic mapping [23].  Some operators measure the diameter of the pulmonary venous ostia to select the circular mapping catheter, while others measure the PV flow Doppler to rule out pulmonary vein stenosis. Although postablation increased PV flow velocity has not been shown to predict pulmonary stenosis, some operators get concerned if the PV flow velocity exceeds 100 cm/sec and is significantly increased from the baseline. During the ablation, ICE helps to identify complications like pericardial effusion, microbubble formation due to tissue over heating as well as thrombus formation on the sheath and catheter. Left atrial-esophageal fistula remains a dreaded complication of atrial fibrillation ablation. ICE helps to define the anterior esophageal-posterior left atrial interface, and can guide changes in the energy-delivery strategy to protect the esophagus from damage during ablation and allow for safe lesion delivery in closer proximity to the esophagus [24,25]. Advances in intracardiac echocardiography include creation of accurate, real time three-dimensional ultrasound geometries that may obviate the need for pre-procedure CT/MRI imaging for catheter ablation of atrial fibrillation [26].

## Three-dimensional rotational angiography

**Figure 7:**
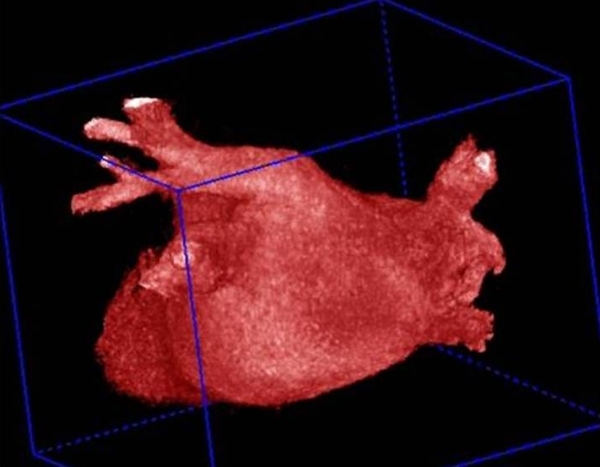
Reconstruction of the left atrial and pulmonary venous anatomy by 3 D rotational angiogram.

While pre-procedure CT/MRI images provide visualization of the left atrium and the pulmonary veins and facilitate integration with the electroanatomic mapping systems, a significant drawback is the time lag to the actual procedure. There may be temporal changes in the size and location of the anatomical structures between the time of image acquisition and ablation. This limitation can be overcome with the use of a real-time three-dimensional rotational angiography (3DRA) in the electrophysiology laboratory for reconstruction of LA and PV as well as the surrounding structures like esophagus (Figure 7) [27,28]. The principle of 3DRA is similar to the CT scan where images acquired from different angles are reconstructed to general a three-dimensional image.  The C-arc X-ray system is rotated over 240 degrees (120 degrees right anterior oblique to 120 degrees left anterior oblique) during the opacification of the pulmonary veins and the left atrium with a contrast medium, either injected directly into the left atrium or in the right atrium or pulmonary arteries and waiting for it to pass through the lungs [29].  The esophagus can be opacified using a barium paste prior to the image acquisition.  The 3D images of the left atrium, pulmonary veins, esophagus and the other surrounding structures can be segmented and registered with the fluoroscopy to serve as a roadmap for ablation [30]. The main advantages of 3DRA includes more accurate representation of the left atrial anatomy, as it is performed immediately prior to the procedure and is not affected changes in volume status of the patient between the day of imaging and the day of procedure. The radiation exposure from 3DRA is less than the CT scan. In case of patient movement, the re-registration is quick and accurate. In comparison to the 3D electroanatomical systems, there is a theoretical concern for increased radiation exposure. Suprisingly, in a recently published randomized study of 3DRA versus electroanatomic mapping system (CARTO) used during atrial fibrillation ablation, the radiation exposure, procedural times and clinical outcomes at 10 months were similar in the two groups [31]. However the use of contrast makes it a less appealing option for patients with heart failure or renal failure. In addition, the technique is sensitive to patient movements during the study period. 3DRA also has the potential to eliminate the need for pre-procedural CT/MRI and the radiation exposure is less than the CT scan. However further refinements are needed before it can be widely adopted. These include incorporation of respiratory and cardiac motion compensation and the ability to display electrogram data on the 3D shell (activation timing, scar and voltage maps, and dominant frequency).

## Summary

Catheter ablation of atrial fibrillation involves pulmonary vein isolation and left atrial ablations. By integrating the CT and MRI images of the left atrium with electroanatomic maps, the electrophysiological navigation systems represent a significant advancement in the field. They enable accurate, real-time navigation with detailed anatomical mapping for the ablation procedure. They enhance the accuracy of lesion delivery, reduce fluoroscopy time and procedural duration and improve the clinical outcomes. Use of intracardiac echocardiography has also evolved over the past few years to provide additional tool to improve the safety and outcomes when used with the electroanatmomic mapping systems.  On the other hand, 3D rotational angiography holds the promise of a emerging as a stand-alone imaging method to guide atrial fibrillation. However further improvements in this modality are needed to make it more widely adopted.  Each mapping/imaging system has its own advantages and limitations and its use is dependent on the skill and preference of the operator. However all of them continue to evolve and hold the promise for facilitating individualized approaches for catheter ablation of atrial fibrillation in the future.
